# Neighborhood Deprivation, Financial Hardship, and Decrements in Kidney Function Across a Decade

**DOI:** 10.1093/geroni/igaf122.4413

**Published:** 2025-12-31

**Authors:** Agus Surachman

**Affiliations:** Drexel University, Philadelphia, Pennsylvania, United States

## Abstract

Neighborhood factors are critical determinants of accelerated renal aging, an important risk for chronic kidney disease (CKD), a pressing public health problem. Little is known about whether financial hardship plays an important role as an individual-level pathway in the association between neighborhood factors and accelerated renal aging. This analysis utilized data from 653 (M age = 54, SD = 9.5; 55% female; 79% NH white) participants who completed the biomarker data collections in both wave 2 and 3 of the Midlife in the United States (MIDUS) study. The neighborhood factor is based on the national level Area Deprivation Index (ADI), a validated neighborhood disadvantage score based on neighborhood socioeconomic factors (1=least disadvantage, 100=most disadvantage). Financial hardship includes material (e.g., lower income to poverty ratio), psychological (e.g., perceived difficulty paying bills), and behavioral (e.g., sold possessions to meet needs) domains. The main outcome was absolute change in creatine-based estimated glomerular filtration rate (eGFR) across a decade. Analyses were adjusted for age, sex, education, and health-related factors (smoking, obesity, hypertension, and diabetes status). Participants living in a highly deprived neighborhood (third percentile or higher) showed faster decrements in eGFR (Est=3.05, SE = 1.42, p<.05). Financial hardship was also associated with faster decrements in eGFR (Est=3.74, SE = 1.19, p<.01). Indirect effect analysis indicated that higher financial hardship was significant pathway through which neighborhood deprivation was linked to faster decrements in eGFR (Est=0.92, SE = 0.39, p<.05). Neighborhood deprivation is a critical risk factor for accelerated renal aging, highly likely due to the higher experience of financial hardship.

